# Exploring short video apps users’ travel behavior intention: Empirical analysis based on SVA-TAM model

**DOI:** 10.3389/fpsyg.2022.912177

**Published:** 2022-07-22

**Authors:** Cheng Wang, Wenjing Cui, Yating Zhang, Huawen Shen

**Affiliations:** ^1^Business School, Nanfang College Guangzhou, Guangzhou, China; ^2^College of Geosciences and Tourism Management, Hanshan Normal University, Chaozhou, China; ^3^China Center for Special Economic Zone Research, Shenzhen University, Shenzhen, China; ^4^Faculty of International Tourism and Management, City University of Macau, Taipa, Macao SAR, China

**Keywords:** short video apps, TAM, eTrust, travel intention, eWOM

## Abstract

Social media had made significant effect on the tourism and hospitality industry. Among diverse types of social media platforms, short video apps (SVA) represented by TikTok or Douyin had brought great changes to the tourism industry. As new mobile technology platform, short video apps had changed the way for user to obtain travel information, make traveling plans and share the travel experience. Considering the new technology of SVA and the influence in tourism, this research aims to explore the SVA users’ behavior intentions and the adopting of SVA for making travel decision. Therefore, the new SVA-TAM model is proposed based on the technology acceptance model (TAM), including two new variables: electronic word of mouth (eWOM) and electronic trust (eTrust). An online survey was conducted to short video apps users. PLS-SEM was implemented for data and structural equations analysis of the final obtained 302 samples. In terms of the relationship between variables, this study found that user perceptions of SVA on usefulness and ease of use are powerful predictors of attitudes toward using SVA for travel planning, which maintains consistency with the outcome of previous TAM studies. Additionally, eWOM and eTrust positively influence user attitudes toward using SVA for travel planning even for destination decisions. Therefore, the short video apps should be taken into consideration for tourism marketing and destination branding owes to the effect on the potential users’ behavior intentions.

## Introduction

Social media has a noteworthy role in numerous angles of tourism, usually including tourism knowledge retrieval, tourism destination decision making, tourism brand promotion, and establishing travel contact with users (Zeng and Gerritsen, 2014). Social media includes social networking sites; video, audio, and photo sharing websites; blogs; apps (location-based social communication); online gaming; and so on. Numerous researchers have examined how social media affects the decision making process. For example, Kim et al. (2017) took Sina blogs as a case study to explore the role of literal and non-literal key indexes of tourism data quality in shaping users’ destination image on social media. Önder et al. (2019) proved the usefulness of Facebook big data. It is evident that researchers endeavor in the area of social media to develop an effective method for destination image branding and customer behavior stimulating.

For video sharing platforms, Stankov et al. (2019) explored YouTube video clips and revealed the user generated content (UGC) characteristics for destination branding. YouTube is generally believed to be useful as a promotional communication tool that offers users the opportunity to hunt for activities, to read online reviews, and to look for help and suggestions about tourism destinations. In addition to videos on YouTube, there is a new emerging video sharing method: short video apps (SVA). TikTok is one of the most representative applications in China, also called Douyin, which first appeared in September 2016. TikTok allows users to create 15 s videos with audio.

Studies investigating the influence of social media, especially short video apps, on destination choice or user behavior intention were previously in an exploratory stage. Nowadays, destination marketing organizations are increasingly facing the use of digital images or videos, but in order to gain insights into visitors’ views on their destinations or their favorite topics, non-text content is difficult to understand. Regarding tourist destinations with photos, Picazo and Moreno-Gil (2019) examined projected images of destinations in the form of photos. Photo sharing platforms such as Instagram and Flicker generate a large number of images with expanding meanings and insights had been omitted to some degree. When it comes to the short video apps, which contain more vivid content with photography skills, diverse subjects, differentiated scenes, and unique audio, previous studies on the text analysis fail to understand the expression for the user-generated contents and the word of mouth on SVA.

Internet users employ social media tools to share their experiences during their travels and after the holiday (Wong et al., 2019). Social media, especially in the reputation area, has a significant impact on the final decision of vacation plans (Fotis et al., 2012). However, current knowledge cannot explain the relevant factors that determine the use of SVAs in specific tourist locations. Do the short video apps stimulate users’ travel behavior intentions?

This research concentrates on filling the gap to exam the underlying factors of short video apps which affect user travel behavioral intention to destination in the context of tourism industry. From a technological perspective, to assess the adoption of SVA for travel planning is affected by two factors: perceived usefulness and perceived ease of use. From electronic trust perspective, to assess the extent to attitude toward using SVA for travel decision is influenced by eTrust on user generated content and eTrust in user generated content provider, respectively. Also, this study examined the relationship between eWOM and attitude toward adopting SVA for travel planning. Based on SVA-TAM model to explore factors which are beneficial to improve the effectiveness of tourism branding and marketing in the social media circumstance and implement related strategies.

## Literature review and proposed model

### Social media and short video app

Short video chip refers to the length of the video chip in seconds. It mainly relies on mobile smart terminals for rapid shooting, editing and uploading, and can be used in social media. Short video app is an application that provides a platform for sharing music and animation effects of short video clips.

The first mobile short video platform, Viddy, was introduced in April 2011(Muchmore, 2013). Users could use the platform to record, embellish, and edit videos. On Android and IOs phones, SVA is very easy to download apps from the app store which are one of the most suitable applications for disseminating information and content. There was an explosive growth in user size, and the mobile short video field became one of the key segments of industry concern.

Video clips’ dissemination through SVAs can have widespread impacts on interest in scenic spots, ancient buildings, customs, and cultures, particularly when these videos have a far reach (Song et al., 2021). The key opinion leaders uploading videos on SVAs form the public assessment, communication for tourism destination expectation, and the initial impression for travel information. User generated videos on SVAs present foods, events, scenic spots, and so on (Wang, 2021).

### Technology acceptance model

The proposal of TAM provided an important basis for interpreting the acceptance and usage of new technologies (Davis, 1989). This model has been verified in many different technology application industries for more than 20 years, and was considered to be a model that stood the test of time for predicting and explaining behaviors in various fields.

**FIGURE 1:**
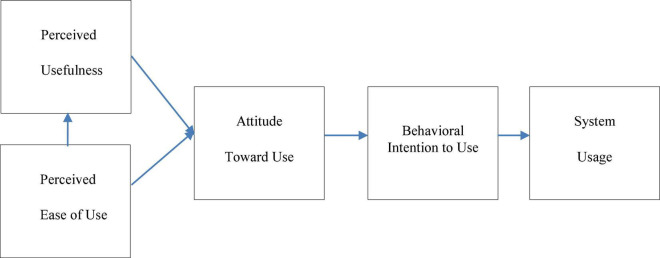
The original technology acceptance model (TAM).

This model is generally based on social psychology theory, especially the theory of reasoned action (TRA) (Fishbein and Azjen, 1975). Beliefs affect attitudes, and attitudes lead to intentions. Therefore, TRA stipulates that behavioral intentions are produced. Corresponding to this, Davis (1989) introduced the following structures in the original TAM (Figure 1): perceived usefulness (PU), perceived easy-to-use (PEOU), attitude, and intention to use behavior. Among them, PU and PEOU constitute the end user’s belief in technology. Therefore, PU and PEOU can predict users’ attitudes toward technology and thus predict their acceptance of technology.

Many scholars had extended the original TAM. There are three types of extended TAM. The first is applied to new environments, such as new technologies (such as information systems and mobile apps) (Peng et al., 2012; Huang et al., 2016), new user groups (such as education, banks and consumers) (Kaplanidou and Vogt, 2006; Kim et al., 2008). The second type adds new structures to expand the scope of theoretical mechanisms described in TAM (Kim et al., 2009; Peng et al., 2012; Ayeh et al., 2013). The third is to introduce new variables into the TAM model and expand the theoretical boundary of TAM to better enhance its interpretation ability (Wöber and Gretzel, 2000; Morosan and Jeong, 2008; El-Gohary, 2012).

In this paper, we explore the usage of short video apps for travel planning and decision making. It is necessary to extend the model of TAM to examine the factors that influence user behavior. Therefore, we looked for journals related to social media to form a new SVA-TAM model for the tourism industry.

### Theoretical framework

**FIGURE 2:**
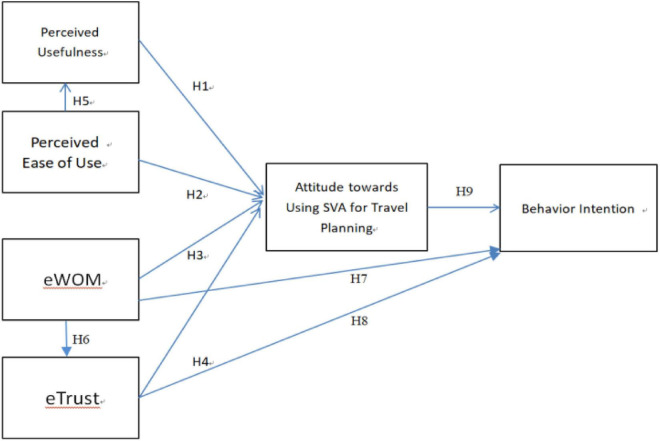
The research model.

This article mainly focuses on short video platforms, considered information exchange technology. Because the interaction between users reflects the characteristics of social networks and the importance of social influence, this research expands the content of TAM by including two constructs: eTrust and eWOM. The development of SVA-TAM (Figure 2) is mainly driven by theory, and its components are derived from previous studies and have been selected to achieve the aims of this article.

#### The effect of perceived usefulness on attitude toward using short video apps for travel planning

Davis (1989) defined perceived usefulness as people thinking that using a specific system can improve their work. Several studies have confirmed that this factor was crucial in the blossoming of hotel and tourism technology (Morosan and Jeong, 2008; El-Gohary, 2012; Ayeh et al., 2013). It was generally believed that if an individual thinks an application is useful for obtaining a specific result, they will use it. For tourists looking for travel content, the availability of shared video platforms determines the role SVAs play in helping them make travel plans and decisions. The relationship between usefulness and attitude has been confirmed by many other studies (Pavlou, 2003; Huang et al., 2016).

In this study, PU reflects the subjective perception that users largely believe that using short video apps can help them better experience on travel planning and decision making. Thus, the following hypothesis is put forward on the basis of the TAM theory:

*H1: PU of SVAs positively influences attitude toward using SVAs for travel planning.*

#### The effect of perceived easy-to-use on attitude toward using short video apps for travel planning, perceived usefulness

Perceived easy-to-use is another important indicator of the Davis model. Davis (1989) proposed that if people thought that an easy-to-use technology can fulfill a task with less effort, everyone will adopt it. When it comes to TAM, the influence of ease of use has been confirmed in previous studies (Wöber and Gretzel, 2000; Kaplanidou and Vogt, 2006; Morosan and Jeong, 2008; El-Gohary, 2012; Ayeh et al., 2013).

Due to different SVA characteristics, accompaniment and visual effects should be considered when measuring PEOU. For example, potential hotel customers need to provide different sources of information when choosing a destination. Users can consider a method that is easier to use and adopt. SVAs are regarded as improving the convenience of obtaining information from the Internet and making travel decisions. PEOU is subjective to a certain extent, reflecting the individual’s feelings about the ease of using an information system. In this study, information systems refer to short video apps, and PEOU is the extent to which users consider social apps to be easy to use. Therefore, the following hypothesis is put forward on the basis of the previous theory:

*H2: PEOU of SVA positively affects attitude toward adopting SVAs for travel planning.*

*H5: PEOU of SVAs positively affects PU.*

#### The effect of electronic word of mouth on electronic trust, attitude toward using short video apps for travel planning, and behavior intention

In this study, eWOM indicates comments whether are positive or negative or likes to video clips on SVA. The definition of eTrust includes the beliefs and expectations of users on the characteristics related to contents and contents providers on SVA. Thus, eTrust is divided into two formative parts: eTrust on UCG and eTrust in UGC provider on SVA.

Scholars in the field of communication and information science have found that digital media resources sometimes lack traditional authoritative indicators, such as the identity or reputation of the author. People who were preparing for travel preferred to obtain travel information from friends, colleagues, and relatives who had previously experienced the destination (Lee et al., 2008b). Dewi Prabandari et al. (2018) added that eWOM information is a major uncertainty that eliminates risks and uncertainties. Compared with WOM communication, eWOM communication had anonymity and lacks incentives.

Mohammed Abubakar (2016) found in a study of 216 tourists in Cyprus that the eWOM of medical tourism is positively correlated with tourism intent and destination trust. Reza Jalilvand et al. (2012) conducted a survey of 264 international tourists and found that eWOM positively influenced the construction of the image of tourist destinations, tourist attitudes, and tourist intentions. Lee and Lee (2009) pointed out that feedback affects people’s attitudes toward brands. Compared with not commenting, highly positive feedback will give people a more active attitude toward the brand; however, negative comments (extreme or even moderately negative reviews) will have a negative effect on brand attitudes.

In the hotel industry, Lee et al. (2008a) found that a considerable number of negative opinions of a restaurant shows that consumers’ negative attitudes toward that restaurant have increased. Sparks and Browning (2011) noted that positive feedback will give hotels more preferential ratings. Similarly, Vermeulen and Seegers (2009) illustrated that a positive evaluation will cause a positive variation in people’s attitude, while a negative evaluation will cause a negative variation in people’s attitude toward a hotel. Therefore, in video clips related to travel information, online comments are more convincing. The above findings gave rise to the following assumptions:

*H3: eWOM influences user attitude toward using SVAs for travel planning.*

*H6: eWOM positively influences eTrust.*

*H7: eWOM significantly influences behavior intention of SVA users.*

#### The effect of electronic trust on attitude toward using short video apps for travel planning and behavior intention

Lin and Ching Yuh (2010) proposed that under normal WOM conditions, trust has a great influence on purchase intention. Similarly, destination trust can stimulate medical travelers’ emotional attachment to a destination, and this attachment can predict consumers’ willingness to sacrifice money to obtain money (Thomson et al., 2005). Surveys showed that tourists were more likely to visit destinations they consider trust-worthy and reliable (Ekinci and Hosany, 2006). Trust has been widely used in ecommerce and other fields in recent years. The credibility of travel-related posts will affect consumers’ purchase intentions (Ang et al., 2001). Consequently, the more trustworthy the user-generated content, the greater its influence on consumer decisions. At this point, eTrust on UGC and eTrust in users will affect the perceptions of tourists. Therefore, by using social media to plan travel routes, tourists can better learn from the experiences of sharing information from acquaintances and relatives. In this way, they have a higher degree of credibility because they can transform travel expectations closer to the final decisions (Yoo et al., 2009).

Because the user is the person who generates and updates all content on the social media independently, and the visitor usually looks for reliable and trustworthy information provided by others, user generated contents are a valuable tourist information resources. These posts, whether text, photos, or videos of tourists’ travel experiences, as well as content and assessments written by short video app users, have improved over time, leading to increased eTrust in social media. It is also well known that the UGC creators offer more reliable and up-to-date information and are regarded as a tourism reference. This information was often needed by other users planning a vacation, so these potential travelers can rely on the experience of others to make decisions.

In view of this, this research proposes the following hypotheses:

*H4: eTrust has a significant effect on the attitude toward using SVAs for travel planning*

*H8: eTrust has a significant effect on the behavior intention*

#### The effect of attitude toward using short video apps for travel planning on behavior intention

Ajzen (1989) defined attitude as the tendency of an individual to react positively or negatively to things, people, systems, or events. Davies defined “use attitude” as “the degree of evaluation influence that a person uses the target system at work,” in the framework of the TAM (Davis, 1993). For this research, the attitude structure implies the use of SVAs for travel planning. For models of customer behavior (Fishbein and Azjen, 1975; Bagozzi, 1981), attitudes establish the relationship with behavior intentions, as has been argued in the literature on information technology and marketing (Gibbons, 2006; Liao et al., 2007; Tao, 2009; Hsu and Huang, 2010; Mamman et al., 2016; Asraar Ahmed and Sathish, 2017; Septiani et al., 2017) as well as tourism and hospitality (Lam and Hsu, 2006; González et al., 2007; Williams and Soutar, 2009; Li et al., 2016); therefore, the following hypothesis is presented:

*H9: Attitude toward using SVAs for travel planning positively affects behavior intention.*

## Methodology

In this research, we construct a theoretical model on the use of short video applications for tourism decision making from two aspects, using PLS software to undertake the structural equation test of the theoretical model, and evaluating the use of these factors by potential tourists and the impact of the intensity and direction.

### Questionnaire development

Electronic trust in this study is regarded as a formative construct. Two formative indicators (eTrust on UGC and eTrust in UGC providers) are examined. For eTrust on UGC, four factors of eTrust on UGC revised from Narangajavana et al. (2017) are adopted; for eTrust in UGC providers, three factors from eTrust in UGC providers revised from Narangajavana et al. (2017) are incorporated and one item from eTrust-Benevolence (Wang et al., 2014) is used.

We adopted the previous questionnaires from Bashir and Madhavaiah (2015) to test users’ attitudes toward using SVAs for travel planning. In our research, PU is a factor to improve decision performance for travel planning through SVAs. PEOU is regarded as the perception of SVA usage. We used the two original indictors of Davis and one indictor from Yang (2013) of variable PU. For the construct of PEOU, two original items from Davis and one from Yang (2013) are utilized.

For the construct of eWOM, four factors derived from Jalilvand and Samiei (2012) are modified for short video apps in the context of tourism. We adopted and modified measurement instruments for behavior intention from studies of hotels and traditional marketing contexts (Kim and Han, 2011; Asraar Ahmed and Sathish, 2017).

After the preliminary design of the questionnaire was completed, in order to ensure the accuracy and rationality of questionnaire in the aspects of question description, item setup, and item size, we invited eight doctoral students of business management, administration, sociology, and other specialties who have used short video apps to seek their opinions on the topic description and topic setup of this questionnaire, and we made adjustments to the initial questionnaire according to their feedback.

After the items for the survey instrument were identified, a questionnaire was designed (see Appendix). The questions were worded to ensure they were concise and reader-friendly and could be translated into Chinese. The draft questionnaire was sent to the expert panel for comments on the content, structure, and format. Revisions were made based on their comments. Then the writer re-translated the Chinese version and invited two professional English translators to make sure that the original meaning was retained. To make the final selection of measurement items, we relied on a pretesting with a sample of 50 online users, prior to the main survey.

### Data collection and sampling

The research validates the proposed model through online surveys. The Internet is an easy way to obtain selected samples. As Poynter (2012) pointed out, “So far, the greatest impact of online market research lies in the quantitative survey and research field of quantity and value.” It is easy to find certain groups through the Internet and allow respondents to respond flexibly at their convenience. Two other important factors are the speed and cost-effectiveness of media delivery (Poynter, 2012). Therefore, in empirical research, the Internet has become one of the most important data collection tools.

For statistical sampling strategy, simple random sampling was implemented, which randomly selects subjects from the target population so that subjects have an equal opportunity to participate. The sampling method is not affected by selection bias or classification error.

A web-based survey was performed to collect empirical data for users who had used social media platforms in China. The survey was distributed via multiple channels appropriate for the target users (social networks such as^1^blogs, travelers’ communities, WeChat, etc.), where the objective of the study was posted together with a link to the webpage that hosted the questionnaire. Inclusion criteria were that the persons had to have access to the mobile internet, were adults (>18 years old), were able to understand Chinese, and were willing to give informed consent to participate. On receiving and clicking the survey link, the participants accessed information about the survey purpose, which included agreement information. After they agreed, they filled in filtering question, and then a series of questions appeared. The participants had to answer each questions in order to proceed to the end. Those who received the survey linkage were also asked to send it to others on their “friends” lists of social media platform. Due to the wide spread of social media, the survey was expanded to many provinces of China covering the different cities of SVA users.

The population of the study comprises individuals who regularly use SVAs for traveling and who have access to the Internet. It is necessary to obtain a sufficient sample size to ensure the precision of the statistical estimation and sampling representation. According to Osterlind et al. (2001), a sample of 300 cases is appropriate to produce stable factor solutions. Dispensing with the view of fixed sample size, some researchers use a flexible method of using the ratio of respondents to items to decide the sample size. Such a ratio could be 10:1 or 5:1. Hair (2009) argued that 10:1 was too restrictive. Therefore, a sample with a 10:1 ratio of respondents to items of measurement was used, being more suitable for multivariate analyses.

### Methods of data analysis

When the research is aimed at expanding existing theories and determining key structures, partial least squares SEM (PLS-SEM) analysis is also preferable to the selected standard proposed by Hair et al. (2011).

Specifically, this study used SmartPLS3.0. The PLS allows simultaneous hypothesis testing, can measure single items and multiple items, and can use formative and reflective indicators (Lohmöller and Lohmöller, 1989; Zelditch et al., 2012). The reason lies in the adoption of PLS as follows: as an exploratory or extension of TAM, the PLS-SEM was appropriate for exploratory research. PLS did not require large sample sizes and normal data. In this study, the data analysis procedures could pay little attention to the normality of the sampling data. The construct of eTrust was the formative one. Considering the whole structural model, the formative variable was a part of the model dealing with reflective variables. PLS-SEM should be used for the data analysis.

## Findings and discussion

### Demographic profile of respondents

Table 1 depicts the demographic data of the survey. The total number of respondents is 817. After the first selective question, whether the user had used short video apps, 613 responses were obtained. Then, with the second selective question, whether the user had been to a destination recommend by SVAs, we gathered 329 questionnaires. After the data examination, the valid sample is 302 in the end. In the main survey, the samples indicated the distribution across gender as 38.41% male and 61.59% female. About 57% fell in the age group of 18–23. Most of the respondents are students of that age period. From the perspective of TikTok’s overall population portrait, the ratio of men to women is more balanced, the target group index (TGI) is between 18 and 35 years old, and users are from first-tier and third-tier cities. Among male users aged 30–35, TikTok’s male and female portraits have a higher degree of preference, while among female users aged 18–29, the preference is higher. For the education level aspect, most respondents hold a bachelor’s degree (69%). The distribution of education level is a shuttle structure, with 2% having less than high school and 3.3% having a doctorate at the poles. Among the top 10 areas, the areas with SVA users with the most distribution were Guangdong, Henan, and Shandong provinces, while Zhengzhou, Xi’an, and Kunming were the cities with the most distribution. For the demographic data, the obtained distribution of users is consistent with the report from TikTok. In China, the south and east area occupies the large SVA market share with users who are potential tourists using SVAs for travel planning.

**TABLE 1:** Demographic profiles of respondents.

Characteristics	Frequency	Proportion
**Gender:**		
Male	116	38.41%
Female	186	61.59%
**Age:**		
18–23	172	56.95%
24–29	42	13.91%
30–35	57	18.87%
36–41	21	6.95%
42–47	8	2.65%
48–53	0	0.00%
More than 54	2	0.66%
**Education:**		
Less than secondary/high school	5	1.66%
Completed college degree	46	15.23%
Bachelor’s degree	209	69.21%
Master’s degree	32	10.60%
Doctorate degree	10	3.31%
**Distribution:**		
North China: Beijing, Tianjin, Hebei, Shanxi, Inner Mongolia	17	5.17%
Northeast: Heilongjiang Province, Jilin Province, Liaoning Province	10	3.04%
East China: Shanghai, Jiangsu, Zhejiang, Anhui, Jiangxi, Shandong, Fujian, and Taiwan	47	14.29%
Central China: Henan Province, Hubei Province, Hunan Province	22	6.69%
South China: Guangdong Province, Guangxi Zhuang Autonomous Region, Hainan Province, Hong Kong Special Administrative Region and Macao Special Administrative Region	205	62.31%
Southwest: Chongqing, Sichuan, Guizhou, Yunnan and Tibet	13	3.95%
Northwest: Shaanxi Province, Gansu Province, Qinghai Province, Ningxia Hui Autonomous Region, Xinjiang Uygur Autonomous Region	15	4.56%

### Assessment of reliability and validity

Table 2 shows that the sum of the overall reliability of the five variables is greater than 0.8. All Cronbach’s alpha values are greater than 0.8. Therefore, the constructs obtain satisfactory internal consistency.

**TABLE 2:** Composite reliability of the major constructs.

	Cronbach’s alpha	Rho_A	Composite reliability	Average variance extracted (AVE)
ATU	0.935	0.935	0.953	0.836
BI	0.914	0.915	0.939	0.795
ETC	0.919	0.919	0.943	0.805
ETP	0.856	0.861	0.902	0.698
EWOM	0.914	0.916	0.939	0.795
eTrust	0.884	0.885	0.945	0.896
PEOU	0.853	0.864	0.910	0.772
PU	0.930	0.930	0.955	0.877

According to Table 3, the indicators of each construct have higher loading values on their own construct than on other constructs. Thus, the results proved a satisfactory performance of discriminant validity.

**TABLE 3:** Discriminant validity test by Fornell and Larcker criterion.

	ATU	BI	EWOM	eTrust	PEOU	PU
ATU	0.9143					
BI	0.8472	0.8918				
EWOM	0.8022	0.7845	0.8916			
eTrust	0.7993	0.7862	0.735	0.9463		
PEOU	0.7466	0.7427	0.7534	0.6719	0.8785	
PU	0.8204	0.7705	0.826	0.7454	0.7443	0.9362

In a more recent context, the discriminant validity needs to be verified through another criterion: the heterotrait–monotrait ratio of correlations. The ratio must be significantly less than 0.85 to prove the validity (Henseler et al., 2016). Table 4 indicates that this criterion is satisfied in this study.

**TABLE 4:** The heterotrait–monotrait ratio of correlations criterion.

	ATU	BI	EWOM	eTrust	PEOU	PU
ATU						
BI	0.849					
EWOM	0.838	0.839				
eTrust	0.837	0.834	0.816			
PEOU	0.799	0.783	0.829	0.770		
PU	0.840	0.837	0.846	0.821	0.824	

### Results of the structural model

The repeatability estimation of the bootstrap method was used to create an empirical sampling distribution for each model parameter, and its standard deviation was used as a proxy for the empirical standard deviation of the parameter. The obtained path model coefficients constitute a bootstrap distribution, which can be used as an approximate value of the sampling distribution. All samples were analyzed by PLS-SEM, and it was found that each channel model coefficient had a standard error. Table 5 lists the path coefficients and test results.

**TABLE 5:** Path coefficients and test results.

Hypotheses	Paths	Path coefficients	*T* statistics	*P*-values	Remarks
H1	Perceived usefulness → attitude	0.294	2.863	0.004	Supported
H2	Perceived ease of use → attitude	0.155	3.028	0.002	Supported
H3	eWOM → attitude	0.2	2.42	0.016	Supported
H4	eTrust → attitude	0.329	6.331	0.000	Supported
H5	Perceived ease of use → perceived usefulness	0.744	20.309	0.000	Supported
H6	eWOM → eTrust	0.736	22.716	0.000	Supported
H7	eWOM → behavior intention	0.159	2.699	0.007	Supported
H8	eTrust → behavior intention	0.172	3.622	0.000	Supported
H9	Attitude → behavior intention	0.623	10.704	0.000	Supported

Hypothesis 1 is that the usefulness of SVAs will positively affect people’s attitudes toward using SVAs for travel planning. It can be seen from the output of PLS that this relationship is significant (*p* = 0.006 < 0.01). This coefficient is 0.291, indicating that the more useful SVAs are, the more positive one’s attitude is toward using SAVs for travel planning. Thus, this hypothesis was supported.

Hypothesis 2 is that the ease of use of SVAs can easily affect users’ attitudes toward adopting SVAs for travel decision making. The path coefficient is 0.159, where *p* = 0.003 < 0.01. Therefore, there is a clear connection between the ease of using SVAs and the attitude toward their adoption for travel planning. Hypothesis 2 was supported.

Hypothesis 3 was related to the eWOM and attitude toward adopting SVAs for travel decision making. The path coefficient is 0.201, *p* = 0.015 < 0.05. There exists a positive connection between eWOM and attitude when using SVAs for travel planning. Assumption 3 is confirmed.

Hypothesis 4 is related to relationships between eTrust and attitude toward adopting SVAs for travel decision making. The path coefficient is 0.328, *p* < 0.001. Hence, Hypothesis 4 was supported.

Hypothesis 5 related to the relationship between PU and PEOU on SVAs. The path coefficient is 0.744 with *p* < 0.001. Research has found that PEOU has a significant impact on PU.

Hypothesis 6 focused on whether eWOM has an influence on eTrust. The path coefficient is 0.736 with *p* < 0.001. Therefore, the eWOM makes a significant contribution to eTrust. Hypothesis 6 was supported.

Hypothesis 7 proposed that eWOM affects the behavior intention of using SVAs for travel planning. The path coefficient is 0.157 with *p* = 0.0076 < 0.05. Thus, Hypothesis 7 was supported.

Hypothesis 8 concerned the relationship between eTrust and behavior intention. The path coefficient is 0.176 with *p* = 0.0002 < 0.001. Thus, Hypothesis 8 was supported. Hypothesis 9 related attitude and behavior intention. The path coefficient is 0.620 with *p* < 0.001. Therefore, the attitude toward using SVAs positively influences the behavior intention of travel planning.

In summary, the findings statistically supported all the hypotheses.

The R-square is a statistic that represents the variance ratio of the dependent variable. One or more independent variables in the regression model are used to explain the dependent variable. Correlation refers to the strength of the relationship between the independent variable and the dependent variable, while *R*-square refers to the degree to which the change in one variable affects another variable. Thus, if the *R*-squared value of the model is 0.50, then the model input could interpret that the observed change can be approximately a half.

Table 6 illustrates the result of the influence size *f*^2^ of each exogenous variable. Based on the point of Hair et al. (2014), the effect size *f*^2^ represents the contribution of exogenous variables to the *R*-squared value of endogenous variables. The three values of 0.02, 0.15, and 0.35 represent small, medium, and large effects, respectively.

**TABLE 6:** Predictive relevance and effect size.

Endogenous variables	*R* ^2^	Exogenous variables	*f* ^2^
eTrust	0.542	eWOM	1.185
PU	0.554	PEOU	1.242
ATU	0.781	EWOM	0.047
		eTrust	0.191
		PEOU	0.041
		PU	0.102
BI	0.812	ATU	0.536
		EWOM	0.044
		eTrust	0.052

### Results of multiple mediating effects

The study followed the method recommended by Hair et al. (2017) to test multiple mediation effects. According to their description, mediation is divided into two types of non-mediation and three types of mediation. According to Table 7, the mediation effect exists in the proposed model: eTrust partially mediates the relationship between eWOM and attitude. Then, eTrust partially mediates the relationship between eWOM and behavior intention. Finally, attitude partially mediates the relationship between eWOM and behavior intention.

**TABLE 7:** Multiple mediation analysis.

Hypotheses and path	Specific indirect effects	Direct effect	Total effect	Types of mediation	Remarks
eWOM to BI via eTrust	0.126***	0.159**	0.56	Complementary partial mediation	Partially supported
eWOM to BI via attitude	0.124*	0.159**	0.56	Complementary partial mediation	Partially supported
eWOM to BI via eTrust and attitude	0.151***	0.159**	0.56	Complementary partial mediation	Partially supported
eWOM to attitude via eTrust	0.242***	0.200*	0.442	Complementary partial mediation	Partially supported

***P < 0.001, **P < 0.01, *P < 0.10, NS, insignificant. ^1^WJX.com

### Formative construct electronic trust interpretation

**FIGURE 3:**
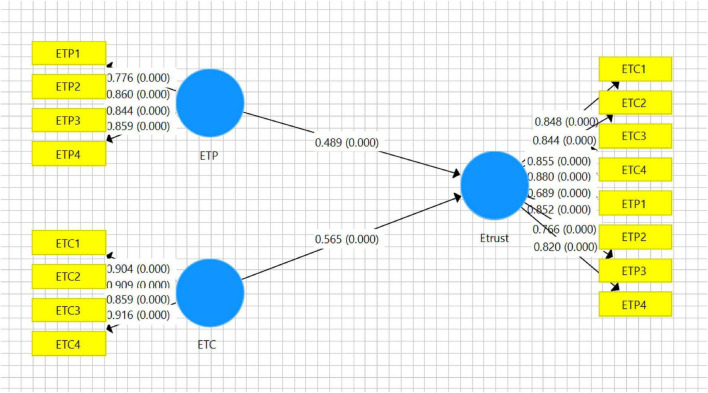
Analysis of formative electronic trust (eTrust) variable.

Partial least squares separately checks the eTrust variable. ETC stands for the eTrust on UGC. ETP stands for the eTrust in UGC providers. The two formative variables eTrust on contents and eTrust in contents providers hold different path coefficients with 0.565 and 0.489 in Figure 3. It indicated that the users prefer to obtain the perceived credibility from SVA through the contents than the providers. So the eTrust on contents of SVA makes significant contribution to the user perception of eTrust on SVA.

## Conclusion

### Discussion

Here, new variables were introduced in TAM to investigate travelers’ intention to use PLS assessments to use short video apps for travel planning. Comparing our results with the previous TAM research, we have some findings to elucidate the adoption of SVAs to influence travel intentions for tourists.

For technological aspect, users’ willingness to use mobile short video apps is positively influenced by users’ perceived ease of use and perceived usefulness. The path coefficient between perceived usefulness and attitude to use is 0.294, which is much higher than between perceived ease of use and attitude to use, which is 0.155.

Perceived usefulness of SVA is regarded as the critical influencer for user’ travel attitude and intention. Users are immersed in huge information flows and often cannot find the content they need quickly and accurately. When users found that the short video platform contained comprehensive and useful information that can save time and costs for them, they prefer to adopt SVA for travel planning. Comparing with the text, pictures, audio format, video clips on SVA make the vivid information presenting format, which was helpful for users to obtain the useful contents to make travel decision.

According to the research conclusions, if the mobile short video platform can provide users with a high perceived ease of use experience, the user’s willingness to use mobile short video platforms will be enhanced. SVA is easy to share travel experience, upload the video clips, and communicate to users. Therefore, the smoother and simpler the operation of SVA, the greater possibility of using SVA for travel planning increases.

For eTrust aspect, it is generally regarded as a key factor in determining user intentions. This research has taken the first step in the application of SVAs in the tourism industry. This study not only proved the crucial effect of the credibility of information on travel social media, but also increased knowledge by deeply analyzing two types of eTrust (eTrust on UGC and eTrust in UGC providers). In this study, two formative variables, eTrust on contents and eTrust in content providers, hold different path coefficients of 0.565 and 0.489, respectively. This indicates that the users prefer to obtain the perceived credibility from SVAs through the content rather than the content providers. Thus, the eTrust in SVA content has a significant contribution to the user perception of eTrust in SVAs. As for believing in the UGC, laymen may think that they are likely to encounter the same or similar situation described in the comments or pictures in the comments of other visitors. They will also think of destinations or travel locations that can provide what they want. So the content looks more like suggestions rather than ordinary content. According to Oklobdžija and Popesku (2017), the trustworthiness of travel-related videos will influence users’ travel decisions. Therefore, the higher the trustworthiness of the user-generated content, the more user decisions are made. As for trusting UGC providers who are friends, key opinion leaders, or acquaintances, user gained the trust from the personality of providers. In this way, users of SVAs have a higher degree of electronic trust, and there is a greater probability of transformation from travel expectations closer to final travel decisions. This move will reduce the risk of users’ travel decisions (Nezakati et al., 2015).

For eWOM aspect, a systematic evaluation method is adopted to establish a model, and employed an empirical structural equation model to illustrate how the eWOM developed on SVAs affects tourism decision-making intentions. It proved that eWOM can influence intentions in the following ways: (i) direct influence, (ii) mediation through different aspects of eTrust and (iii) mediation through attitude. This research responds to the call of Cheung and Thadani (2010) to establish a theoretical basis for eWOM research and deepen our understanding of how eWOM affects social media adoption intentions for travel planning. Specifically, this is one of the studies on why eWOM has an impact on eTrust and why eTrust has an impact on attitudes and decision-making intentions.

### Theoretical contributions

In theory, this study provides a new direction of technology acceptance research for the comprehensive application of SVA characteristics by proposing a multidimensional model of the determinants of SVA adoption. This model enriches theories by constructing new variables in the combination of the Technology Acceptance Model (TAM) and Planned Behavior Theory (TPB), and applies them to new overlapping environments between tourism and social media. However, traditional consumer behavior has been fully proven by economics and marketing theories, and research has shown that technology-related variables are as important as traditional variables (McKnight et al., 1998). The results of this study indicate that for user behavior researchers, the role of testing uncertainty is crucial in situations where eTrust and eWOM may affect the use of SVAs. Therefore, the current method adds rich and profound insights to the personal response to understanding and adopting SVAs. In addition, the model proposed in this study makes a significant contribution to the emerging social media literature, especially on short video apps.

By adding variables such as eTrust and eWOM to the TAM framework, this study offers to deepen people’s understanding of the role of these two concepts in forming understanding, thereby affecting the attitude and intention of technology use. In addition, the research also provides a theoretical basis for TAM research through the influence of external factors on TAM’s core beliefs (PU and PEOU). The results of this study emphasize how PU and PEOU are formed, and how they affect the acceptance of new information systems (such as SVA). The experimental results also show that the combination of TAM and TPB with other variables is effective, laying a foundation for the integration of other technology acceptance models.

This article develops a theoretical model for how eWOM affects intent through consumer eTrust on content and content providers. Using this model, we can better understand how eWOM on SVAs affects travel decisions. On this basis, this article puts forward a new theoretical basis, using SVAs as a communication platform to study the influence of eWOM on travel decisions. The eWOM on SVAs will directly affect travel decisions and people’s attitudes and trust perception in social media. This theoretical model can explain the phenomena of visiting Internet-worthy locations.

Overall, the proposed model indicates acceptable model fit and explains insights into user traveling decision progress through SVAs in a novel context. In terms of the relationship between variables, we found that the usefulness of perception and the ease of use of perception are powerful predictors of SVAs’ impact on travel planning in accord with the outcomes of previous TAM research. Additionally, eWOM and eTrust should be considered, as this study also proved they were significant predictors of user attitudes toward using SVAs for travel planning even for final decisions. The most influential variable is eTrust, therefore, the higher the trustworthiness of UGC and UGC providers, the more user travel decisions are made by SAV acceptance. PU is the second most influential variable, comparing the SVA function and performance, it is the contents on SVA that have the crucial effect on user travel decision making and destination choice. Attitude is regarded as mediation effect variable of PU and PEOU holding fully mediation and of eTrust and eWOM presenting partially mediation to behavior intention.

### Managerial contributions

The survey results show that if users’ beliefs in eWOM, eTrust, usefulness and ease of use are properly managed, their willingness to adopt SVA for travel planning will increase. Attitudes and beliefs play an important role in users’ intention to use SVAs. Therefore, DMO can focus more on the positive attitude of potential consumers toward SVA destinations. The study has confirmed that trust is one of the most critical factors in perceptions of SVA content and has a more direct impact on travel decisions, indicating the importance of building trust in SVA branding and marketing.

As “tourism theme” or “tourism IP” have become hot topics in the tourism industry, the SVA platform has become an exciting marketing channel for practitioners in the tourism industry. It is the first example of TikTok being used in tourism marketing. TikTok provided an opportunity to create a platform to present images of the current destination and to propose advice for future travel. As always, using an SVA as a travel marketing tool, the key to success is to accurately understand target customers and customize products according to their interests and needs related to the tourism industry. SVAs comprise a rapidly developing platform. To avoid losing interest among the community, real-time marketing has become necessary. In other words, companies must respond quickly and always pay attention to current travel trends. Therefore, tourism or destinations related to the content must not only have “creative video clips,” but also have a novel design that can attract the attention of the target group.

Since COVID-19 broke out, users have been forming a habit to watch SVA videos. Short video app has overtaken instant messaging as the area where people spend the most time online, and it is growing fast. As of December 2021, the customer stickiness of short video users exceeded that of other industries, with a year-on-year growth of 4.7%, and the total time spent using short video app accounted for 25.7% (QuestMobile, 2021).

The SVA is a dual source of entertainment and information. Due to SVA’s innovations in creativity, film, and editing, and a full understanding of what users are interested in watching, there are many opportunities to incorporate SVAs in any industry. Storytelling is an important factor in the successful production of short films, and music is essential for every video clip. Choosing currently popular songs can give destination brands greater appeal to young audiences.

### Limitations

This paper contains an empirical analysis of the factors affecting the willingness to use mobile short video platforms, and obtains some confirmed results, but there may be some shortcomings in the research process.

First, survey samples may have certain limitations. Because of the limitation of objective conditions, time cost, and other factors, the subjects of this study were mainly selected through the authors’ network via electronic questionnaire distribution. Thus, the age group is mainly young people aged 18–30. Although these are the main users of mobile short video platforms at present, expanding the coverage of the sample is conducive to an improved and comprehensive analysis. Follow-up researchers could therefore consider expanding the sample collection to make it more representative.

Second, this study did not make full use of the respondents who were willing to participate. The total number of respondents reached 871. Nearly 258 of them did not use short video apps, so they did not answer the first question. The number of people who used short video apps was 613, and after the second selective question, we obtained 329 valid samples. These respondents filled out a follow-up questionnaire. Because of the difficulty in obtaining information from the respondents, it is possible to improve the relevant items in follow-up work, and also to include questions for the non-users who are willing to participate, such as their reasons for non-utilization.

## Data availability statement

The original contributions presented in this study are included in the article/Supplementary Material, further inquiries can be directed to the corresponding author.

## Ethics statement

Ethical review and approval was not required for the study on human participants in accordance with the local legislation and institutional requirements. Written informed consent from the patients/participants OR patients/participants legal guardian/next of kin was not required to participate in this study in accordance with the national legislation and the institutional requirements.

## Author contributions

CW: conceptualization, investigation, methodology, and writing – original draft. WC: data curation and formal analysis. YZ: responses to reviewers, manuscript revision, and english proofreading. HS: supervision, project administration, writing – review and editing, and validation. All authors contributed to the article and approved the submitted version.

## Conflict of interest

The authors declare that the research was conducted in the absence of any commercial or financial relationships that could be construed as a potential conflict of interest.

## Publisher’s note

All claims expressed in this article are solely those of the authors and do not necessarily represent those of their affiliated organizations, or those of the publisher, the editors and the reviewers. Any product that may be evaluated in this article, or claim that may be made by its manufacturer, is not guaranteed or endorsed by the publisher.
