# Supplementary material for: Exploring short video apps users’ travel behavior intention: Empirical analysis based on SVA-TAM model
Source: Front Psychol. 2022 Jul 22;13:912177. doi: 10.3389/fpsyg.2022.912177 (PMC9355327; doi:10.3389/fpsyg.2022.912177)
Supplement: Supplementary file 1 [file Data_Sheet_1.pdf]

Appendix

A Study of Exploring Short Video Apps Users' Travel Intention

Formal Questionnaire

Dear Sir / Madam:

We would like to invite you to participate in this research project. The purpose of this study is to understand tourist's usage of short video apps for travel planning. This anonymous survey will not collect your personal identity data. There is no right or wrong answer. The information provided will only be used for academic research. No known risks are associated with this project. Thank you for your participation.

Wang Cheng

Ph.D. of City University of Macao

Filter Questions

1. Have you ever used short video apps?

1. □ No (End of Survey) 2. □ Yes

2. Have you ever been to the destination recommended (including: destination, food, culture, customs and scenic spots) by SVA?

1. □ No (End of Survey) 2. □ Yes

Please give the following questions scores about short video apps based on your personal experience(s) considering you have watched the video clips about tourism related contents (destination, food, culture, customs and scenic spots) on short video apps.

Totally disagree =1； disagree =2； partially disagree=3; neutral =4； partially agree=5; agree=6； totally agree =7

	Attributes	1(Totally disagree) – 7 (Totally agree)						
eTrust on content	Comments about the destination on short video apps are true.	1	2	3	4	5	6	7
	Photos or videos of the destination on short video apps match the real situation.	1	2	3	4	5	6	7
	With contents on short video apps, I feel I know what to expect from the destination before I travel there.	1	2	3	4	5	6	7
	I believe that what people have posted about destination on short video apps is reliable	1	2	3	4	5	6	7

eTrust in UGC provider	They recommend me contents (scenic spots, food) that I like through short video apps.	1	2	3	4	5	6	7
	They are honest with the information they posted on short video apps.	1	2	3	4	5	6	7
	When there is something that can be of benefit to me for my travel decision, they tell me on through video on short video apps.	1	2	3	4	5	6	7
	Providers have a good reputation on short video apps.	1	2	3	4	5	6	7
eWOM	I often read other tourists' online travel reviews to know what destinations make good impressions on others.	1	2	3	4	5	6	7
	To make sure I choose the right destination, I often read other reviews.	1	2	3	4	5	6	7
	I frequently gather information from tourists' online travel reviews before I make decision.	1	2	3	4	5	6	7
	Before I travel to a destination, the more likes of online reviews, the more confident I have to choose destination.	1	2	3	4	5	6	7
PU	Using short video apps provides me with information that would lead to better travel decision.	1	2	3	4	5	6	7
	Using short video apps enhances my effectiveness in travel decision.	1	2	3	4	5	6	7
	Short video app is useful for travel decision.	1	2	3	4	5	6	7
PEOU	It is easy to become skilful in using short video apps.	1	2	3	4	5	6	7
	Using accompaniment and effect on short video apps would be easy for me.	1	2	3	4	5	6	7
	I would find it easy to get short video apps to do what I want it to share.	1	2	3	4	5	6	7
Attitude	Using SAV for travel planning is a good decision.	1	2	3	4	5	6	7
	Using SAV for travel planning makes me feel happy and favorable.	1	2	3	4	5	6	7
	When making travel planning or destination choice, SVA is a beneficial tool.	1	2	3	4	5	6	7
	When making travel planning or destination choice, SVA is a valuable tool.	1	2	3	4	5	6	7
BI	I will always use SVA for travel planning.	1	2	3	4	5	6	7

	I would recommend videos and short video apps to friends for travel decision.	1	2	3	4	5	6	7
	I would make travel decision through short video apps.	1	2	3	4	5	6	7
	I would like to go the destination recommend by short video apps.	1	2	3	4	5	6	7

3. How many times have you used SVA for travel decision?

□One

□Two

□Three

□Four

□Five

□ Six

□Seven or above

Demographic Information:

1. Gender:

A. Male B. Female

2. Highest education level

A. Junior middle school or below B. High school C. Bachelor's degree D. Master's degree
E. Doctor degree

3. Age:

A.18 -23 B.24-29 C.30-35

D.36-41 E.42-47 F.48-53 G. more than 54

4. Your career is

A. Students B. Full-time housewives C. Teacher or other education industry

D. Service industry E. Tourism Industry F. Culture Industry

G. Information &Technology H. Others (please specify) _____________

5. Which app of short videos apps do you often use?

A. Tiktok (Douyin) B. Kuaishou C.Weishi D.Snapshot E. Huoshan

6. Where are you from?

North Area: Beijing, Tianjin, Hebei, Shanxi, Inner Mongolia

Northeast Area: Heilongjiang Province, Jilin Province, Liaoning Province

East Area: Shanghai, Jiangsu, Zhejiang, Anhui, Jiangxi, Shandong, Fujian, and Taiwan

Central Area: Henan Province, Hubei Province, Hunan Province

South Area: Guangdong Province, Guangxi Zhuang Autonomous Region, Hainan Province, Hong Kong Special Administrative Region and Macao Special Administrative Region

Southwest Area: Chongqing, Sichuan, Guizhou, Yunnan and Tibet

Northwest Area: Shaanxi Province, Gansu Province, Qinghai Province, Ningxia Hui Autonomous Region, Xinjiang Uygur Autonomous Region
